# Facilitating 1.5T MR‐Linac adoption: Workflow strategies and practical tips

**DOI:** 10.1002/acm2.70073

**Published:** 2025-03-16

**Authors:** Madeline Michel, Zelda Paquier, Christelle Bouchart, Akos Gulyban, Nicolas Jullian, Dirk Van Gestel, Sara Poeta, Nick Reynaert, Philippe Martinive, Robbe Van Den Begin

**Affiliations:** ^1^ Radiation Oncology Department Hôpital Universitaire de Bruxelles (HUB) Université libre de Bruxelles (ULB) Institut Jules Bordet Brussels Belgium; ^2^ Medical Physics Department Hôpital Universitaire de Bruxelles (HUB) Université libre de Bruxelles (ULB) Institut Jules Bordet Brussels Belgium; ^3^ Université libre de Bruxelles (ULB) Brussels Belgium; ^4^ Medical Physics Department Hôpital Universitaire de Bruxelles (HUB) Radiophysics and MRI Physics Laboratory (ULB836) Université Libre de Bruxelles (ULB) Brussels Belgium

**Keywords:** adaptive radiotherapy, MR‐guided radiotherapy (MRgRT), MR‐Linac, workflow optimization

## Abstract

**Background:**

MR‐guided radiotherapy (MRgRT) offers new opportunities but also introduces workflow complexities requiring dedicated optimization. Implementing magnetic resonance linear accelerator (MR‐Linac) technology comes with challenges such as prolonged treatment times and workflow integration issues.

**Purpose:**

We present here our experience and share practical tips and tricks to streamline MR‐Linac implementation, optimize workflow efficiency, and improve coordination.

**Methods:**

The first 150 patients treated with a 1.5T MR‐Linac Unity^®^ at our institution were analyzed. Treatments were assessed based on session recordings, difficulties encountered were identified, and solutions documented.

**Results:**

A total of 726 fractions were delivered, with a mean treatment time of 48 minutes. Key optimizations included standardized operating procedures (SOPs) and structured briefing sheets, leading to reduced delays and improved treatment consistency.

**Conclusion:**

Strategic workflow standardization and optimized communication tools significantly improved the ability to deliver high‐quality, patient‐centered care by streamlining processes and enhancing coordination among team members. These insights provide practical guidance for centers integrating MR‐Linac technology.

## 1 INTRODUCTION

The field of adaptive radiotherapy (ART) has undergone significant changes with the introduction of magnetic resonance guided online ART, by integrating magnetic resonance imaging (MRI) with a linear accelerator (linac), providing better soft tissue visualization and allowing imaging simultaneously to the treatment delivery.^1^, ^2^, ^3^ Moreover, this technology enables daily and online treatment plan adaptation based on tumor and anatomical changes observed within the treatment session (i.e., online ART). Clinical indications for MR‐guided adaptive radiotherapy (MRgRT) are expanding as more clinical evidence and studies focus on this new hybrid machine.^1^, ^4^, ^5^ The magnetic resonance linear accelerator (MR‐Linac) technology offers several advantages, including online ART and subsequently the application of smaller margins to reduce toxicities,^4^, ^5^, ^6^, ^7^, ^8^ as well as extreme hypo‐fractionation treatments.^9^, ^10^, ^11^ Additionally, the real‐time gating enables even more precise adaptive treatments.^12^, ^13^

Conversely, this technology combining an MRI with a linac, results in additional unusual challenges compared to a cone beam computed tomography (CBCT)‐based linac. Among these are MR safety precautions, anatomical and functional MR images acquisition, adaptive decision‐making, online contouring, and re‐planning. Altogether, they result in a significant increase in treatment session time and a complete reorganization and investment of the human resources.^4^, ^8^, ^14^, ^15^ While several studies have reported on the clinical workflows and treatment times associated with the MR‐Linac,^16^, ^17^, ^18^, ^19^ they rarely provide detailed strategies for optimizing workflow efficiency or addressing real‐time challenges encountered during implementation. Our study aims to bridge this gap by presenting practical improvements and unique institutional experiences.

In this report, we present our initial experience with the first 150 patients treated on a 1.5T MR‐Linac Unity® (Elekta Ltd. in Stockholm, Sweden). We believe sharing this information will facilitate the introduction and implementation of MR‐Linac‐based treatment in other radiotherapy centers. We explore the impact of MRgRT on clinical practice, focusing on treatment time and the key factors influencing it. Finally, we present the practical measures we implemented to optimize the daily clinical workflow.

## 2 MATERIALS AND METHODS

After the official training provided by Elekta, we decided to refine it before the first patient by simulating the practical MR‐Linac workflow with healthy volunteers. We conducted our observations and analysis on the MR‐Linac workflow between June 2022 and May 2024 corresponding to the first 150 treated patients. To balance patient comfort and treatment time, the MR‐Linac was only used for hypofractionated treatments up to five fractions.

**FIGURE 1:**
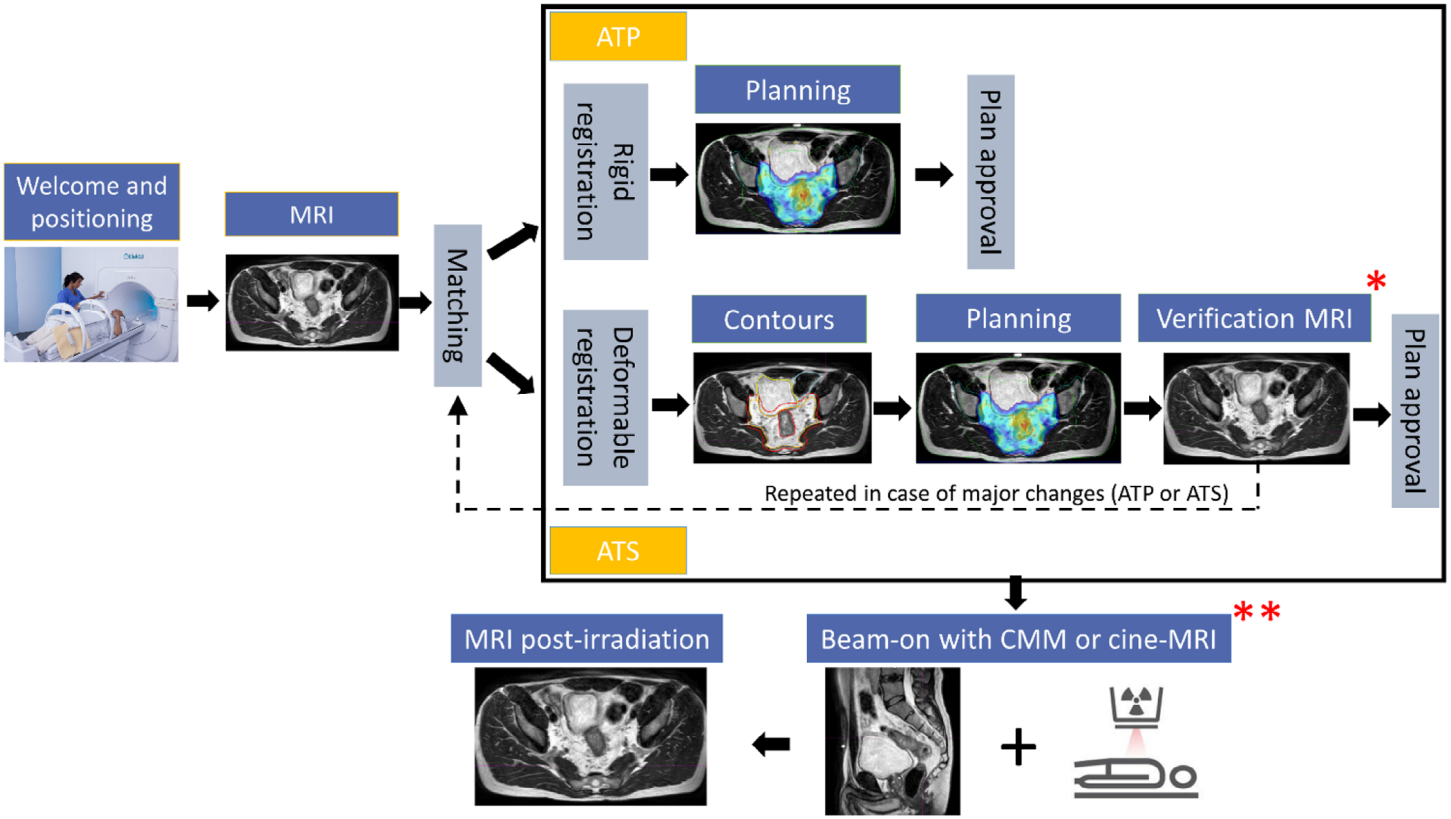
Treatment workflow step by step. The workflow starts with patient welcoming, positioning, and acquisition of a planning magnetic resonance imaging (MRI) for treatment adaptation. The new image is matched to the reference image to determine the adapt‐to‐position (ATP) or adapt‐to‐shape (ATS) pathway. For ATP, rigid registration is performed, and dosimetry is recalculated based on the shift. For ATS, deformable registration is followed by re‐contouring, re‐planning, and a verification MRI to confirm stability before plan approval. Treatment proceeds with cine‐MRI or CMM during beam‐on, and a post‐irradiation MRI completes the process. *CMM includes template validation before plan approval (not used here). **CMM also enables adjustments during beam‐on.

For simulation, most patients underwent two sessions: one on a simulation computed tomography (CT) scanner to calculate electron density and provide a backup option for conventional linac treatment, and another on the MR‐Linac for treatment planning based on MRI sequences.^20^, ^21^ During daily sessions, the Unity system offers two main online adaptive modes: adapt‐to‐shape (ATS) and adapt‐to‐position (ATP). ATS involves recontouring and replanning during treatment to ensure daily adjustments, while ATP shifts the virtual isocenter without recontouring, suitable for cases with stable anatomy^22^, ^23^ (Figure 1). The choice of the adaptive mode is made daily, based on the patient's anatomical situation. Those two approaches can also be combined within the same fraction, particularly if the patient moves during the session or if anatomical changes occur, such as bladder filling causing structural deformation. Elekta Monaco® version 5.51.11 was used as an offline and online treatment planning system, and Mosaiq® version 3.10 as a record and verification system.

**FIGURE 2:**
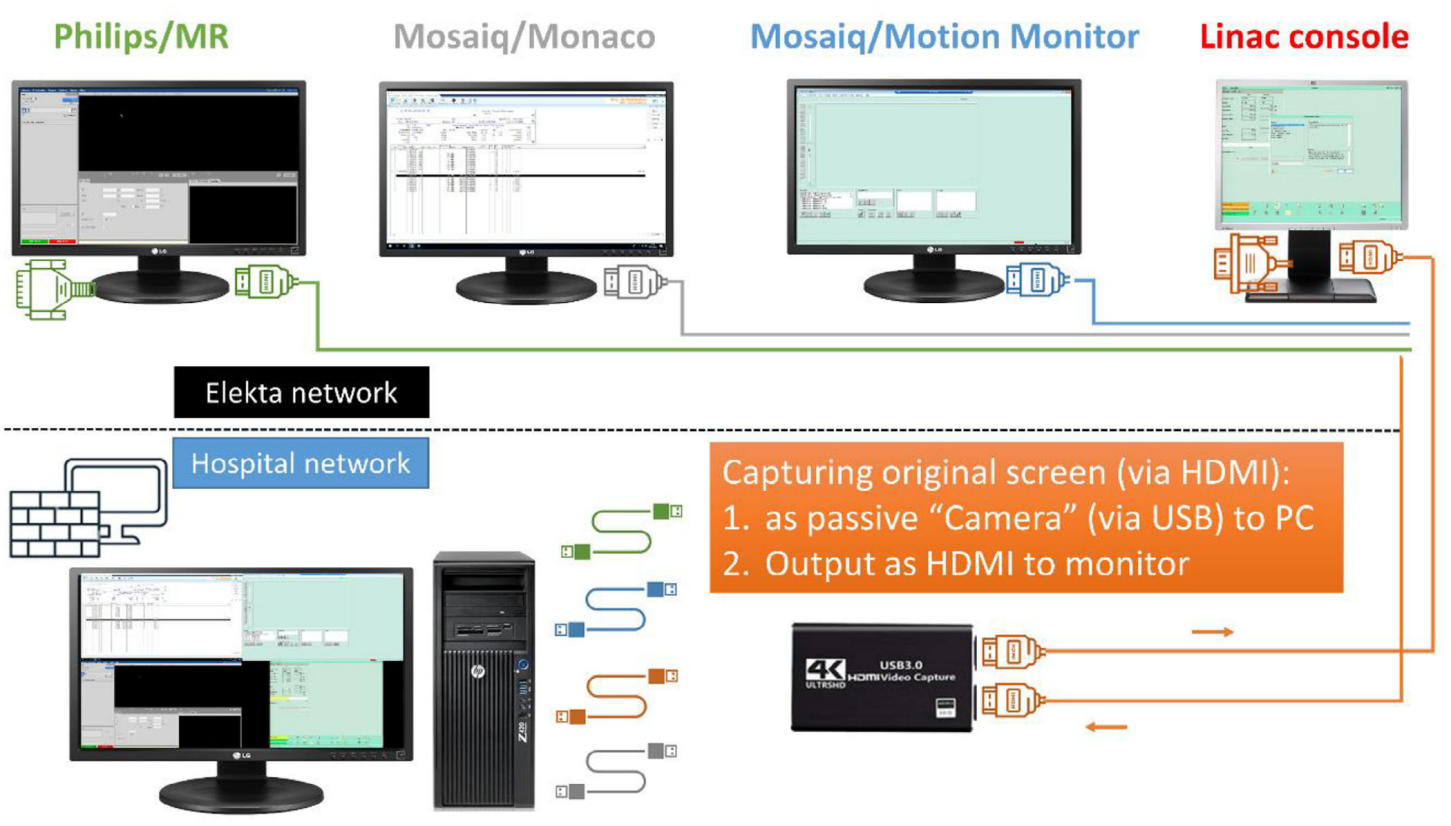
Screen recording set‐up.

To evaluate our practice in detail, we set up a screen recording system of four consoles simultaneously (Philips® imaging console, Monaco/Mosaiq, real‐time imaging screen, linac console) to perform detailed offline analyses, capturing full activity recordings without human interaction (Figure 2). For each treatment session, the recordings were analyzed offline to evaluate the time spent on various steps in a typical MR‐Linac treatment session, including MRI acquisition, matching/adaptation decision, contour adaptation, treatment planning, pre‐beam verification, beam‐on time, and post‐treatment MRI (which was usually started during beam‐on but sometimes lasted longer than beam‐on). We also documented difficulties encountered and their solutions, categorizing these events as either machine‐related or “avoidable steps” (referring to a suboptimal execution of the workflow that could have been avoided if more attention had been paid to certain details).

## 3 RESULTS

In total, 726 radiotherapy (RT) fractions were delivered to the first 150 patients treated on the MR‐Linac, out of which 666 had full‐screen recordings for a complete session time analysis. All patients tolerated the treatment well. The treatment sites included abdominopelvic lymph nodes (*n* = 26, 35–40 Gy/5 or 30 Gy/3), rectum (*n* = 25, 25 Gy/5), pancreas (*n* = 31, 50 Gy/5), liver (*n* = 25, 40–60 Gy/5), prostate (*n* = 35, 40 Gy/5), adrenal (*n* = 4, 35–50 Gy/5), gynecological sites (*n* = 3, 40 Gy/5 or 28.4–30.8 Gy/4), and one stomach (*n* = 1, 30 Gy/5) (see Table 1 for MRI sequences).

**TABLE 1:** MRI sequences used by localization in absolute number.

Localization	T2 3D Tra	T1 3DVaneXD	T1 3DVaneXD SPAIR	b3DVaneXD	b3DVaneXD SPAIR
Liver	4	12	6	–	3
Lymph nodes	8	1	–	13	4
Pancreas	1	–	–	9	21
Prostate	35	–	–	–	–
Rectum	25	–	–	–	–
Others	4	2	–	2	–

*Note*: T2 3D Tra: T2‐weighted 3D imaging (includes T2 3D Tra and T2 3D Tra 2 min). T1 3D: T1‐weighted 3D imaging. b3D: Balanced steady‐state free precession (bSSFP) 3D sequence. VaneXD: Radial MRI sequence designed to reduce motion artifacts, particularly from respiratory or cardiac movement. SPAIR: Spectral Attenuated Inversion Recovery (SPAIR) for fat suppression.

**FIGURE 3:**
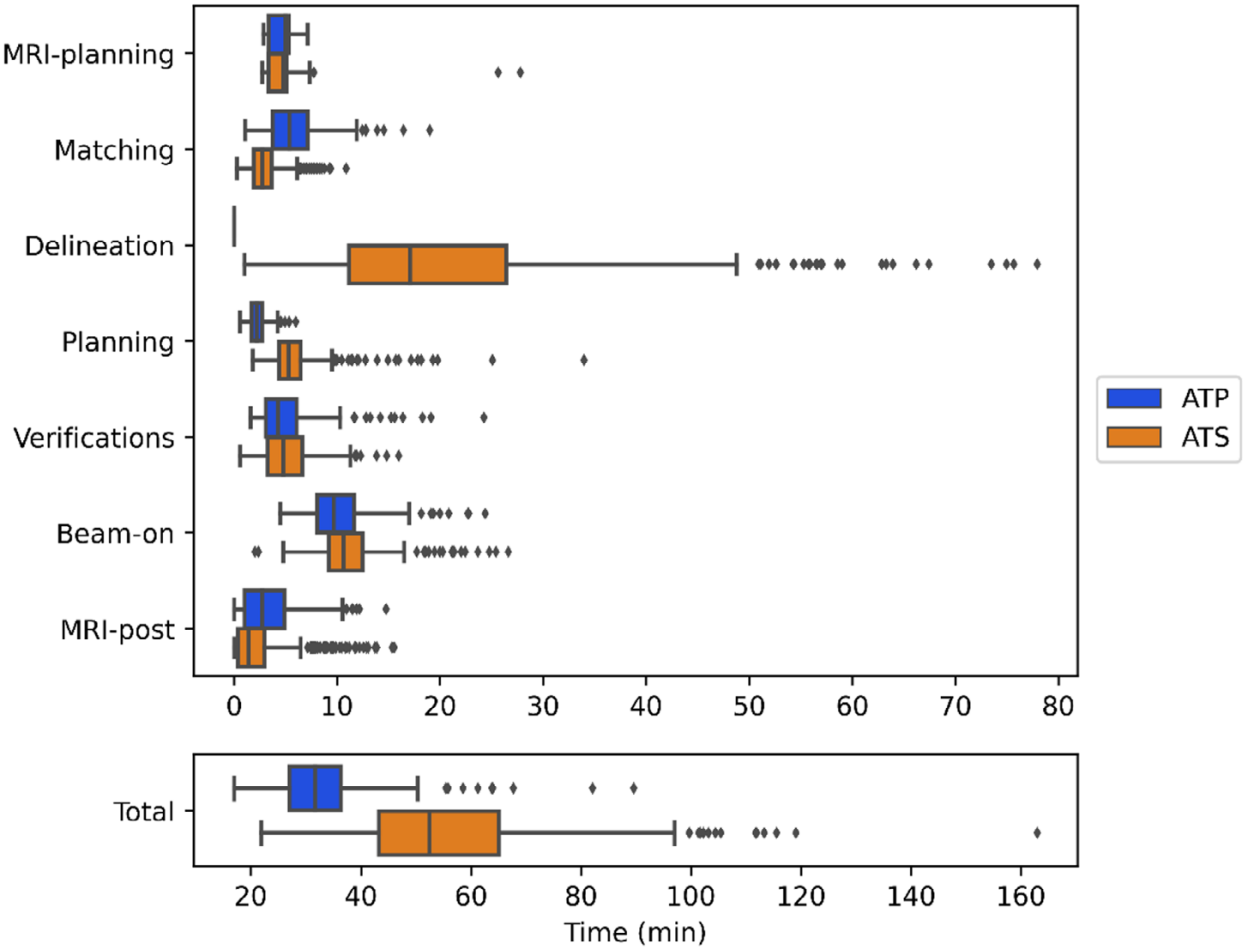
Time distribution (in minutes) per treatment step and per fraction and stratified based on adapt‐to‐shape (ATS) (orange) or adapt‐to‐position (ATP) (blue).

**FIGURE 4:**
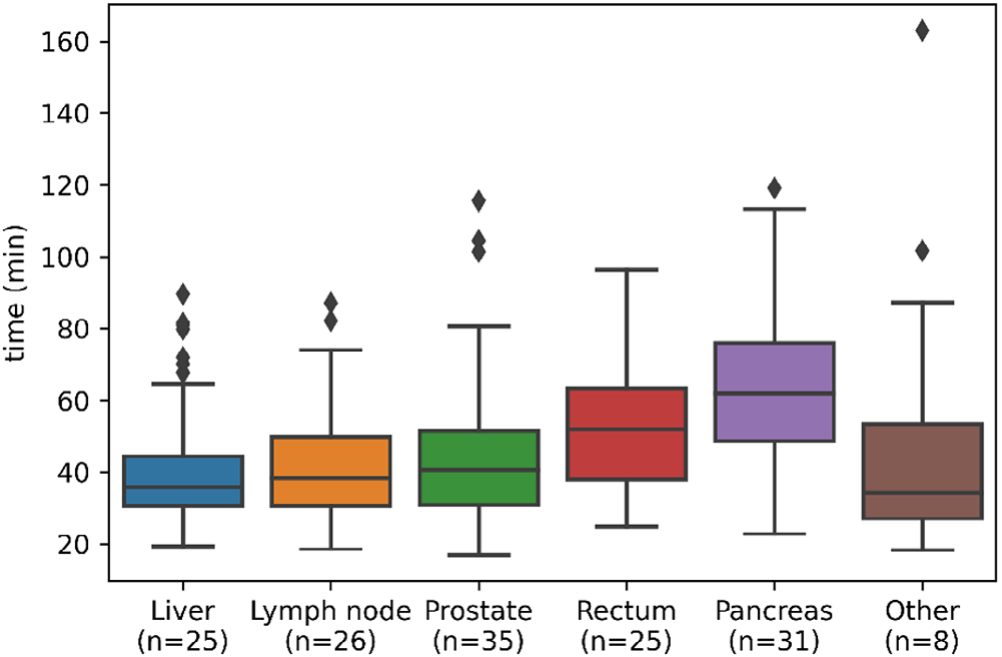
Boxplot of total time (in minutes) spent on the table per pathology.

The mean time spent on the treatment table was 48 min (range 15.1–119.1 min). Of all fractions, 424 were completed using ATS (58.4%) with a mean on‐table time of 53 min, 229 with ATP (31.5%) with a mean time of 32 min, while the remaining 73 combined multiple adaptations (10.1%) with a mean time of 73 min (Figure 3). This included seven completion plans due to beam‐on interruption (four machine‐related, three patient‐related), resulting in approximately a 30‐min time loss. We also stratified treatment time based on tumor site, noting longer treatment times for pancreatic disease (Figure 4).

Machine‐related issues included technical and interconnectivity problems between systems, such as Monaco to Mosaiq connection issues, magnetron arcing incidents and/or magnetron restarts, Mosaiq to Linac interface (NRT) failures, margin structure discrepancies, and various machine connection concerns. Additionally, we identified “avoidable steps” that led to time losses, with some requiring re‐contouring or re‐fusion, preventable through workflow optimization. To address these issues, we introduced mandatory steps in the online workflow, emphasizing tasks like using a “contour ring” structure around the planning target volume (PTV) to reduce contouring time and focus on structures near the tumor. During the online contouring phase, we added a support screen presenting multimodality reference contours from MIM software to accelerate adaptation, especially in challenging areas. We also implemented a “clean all structures” task before recreating margins and validating delineation to avoid misplaced dots of OAR and clinical target volume (CTV), and used crop/margin structures created in advance. Finally, we ensured the removal of “hanging” functional images from the Monaco Online import folder before plan approval. This step is crucial to avoid the crash of Monaco during export if no verification sequence is acquired after a functional sequence, particularly to remove non‐Elekta approved MRI sequences, such as diffusion‐weighted imaging (DWI) sequences (Supporting Information A).

With the increasing number of complex cases referred to our Unity, effective communication of patient care and treatment information became crucial, especially with daily team changes. This led to the development of a digital central communication document per patient, called the “Briefing Sheet”, which is created when the treatment plan is prepared, and updated during the RT series (see Supporting Information B). Key elements incorporated into the briefing sheet included details regarding the imaging acquisition protocol for the treatment session, contouring strategy (e.g., “maintain rigid volume”, “crop within the liver volume”), predefined margins, plan optimization particularities, specific dose constraints, and maximum acceptable longitudinal shifts in cases involving a long PTV (> 14 cm craniocaudally). This sheet also recorded day‐to‐day treatment decisions and specific notes to ensure consistent and effective patient care.

**Tips and tricks** (detailes on how and why in Supporting Information A)

‐ **“Contour ring” support structure to re‐contour efficiently (2–3 cm around PTV)**.
✓ Time gained in contouring.
‐ **Record solutions to previous technical problems (error logbook)**.
✓ Time gained globally (phase‐dependent issue).
‐ **Clean structures before recreating margins**.
✓ Time gained in contouring (or in planning if detected afterward).
‐ **Prepare structures for cropping and margin expansion before online planning**.
✓ Time and quality gained in contouring.
‐ **Empty the Monaco Online import folder if the last imaging before plan approval was a functional sequence**.
✓ Time gained in handling export errors.
‐ **Briefing sheet for each patient (specificities, dose constraints, daily notes, …)**.
✓ Time and quality gained globally (useful in all phases), and better communication.
‐ **Additional screen with patient's baseline contours just above the main screen**.
✓ Substantial time and quality gained in contouring.

## 4 DISCUSSION

Online MR‐guided radiotherapy opens a new era in radiation oncology, offering a promising step toward enhanced precision and improved patient outcomes. However, implementing new technologies like MRgRT in clinical practice presents significant challenges. Based on our first 150 patients, we share key observations and insights to streamline treatment processes and improve workflow efficiency.

### 4.1 Key findings: Treatment timing and workflow efficiency

Treatment timing is a central aspect of MRgRT, as session duration directly impacts patient comfort and intra‐fraction stability. Treatment times on the Unity vary according to numerous parameters and may differ from one center to another. Nevertheless, online ART is a delicate balance to strike between precision gained by improved imaging and adaptation versus precision lost due to longer treatment time. Ruggieri et al. and Werensteijn‐Honingh et al. provided valuable insights into this trade‐off and underscored the importance of improving ATS and ATP procedures to optimize session time while maintaining clinical efficacy.^8^, ^15^ Part of the overall treatment time is associated with our decision to treat with extreme hypofractionation, resulting in an average beam‐on time of 5–12 min, limited by hardware constraints (e.g., dose rate maximized at 480 monitor units per minute [MU/min], step and shoot intensity‐modulated radiotherapy [IMRT]). Our findings align with previous clinical experiences reporting average ATS session times ranging from 38 to 79 min.^1^, ^16^, ^24^, ^25^, ^26^ However, our study extends these findings by providing detailed strategies for optimizing workflow efficiency, such as the briefing sheet, the logbook error, or specific structures prepared in advance. It is challenging to precisely quantify the time saved through these tips, as it depends on the nature of the issue. Additionally, the briefing sheet was quickly integrated, and session times were heavily influenced by the confidence and efficiency of each physician, which improved with experience and the number of patients treated. It is worth mentioning that the Unity system is being updated with motion management technology for tumor gating, which will slightly adjust workflows and timing. These updates may extend beam‐on time while enabling gating and adaptations during irradiation.

### 4.2 Challenges and solutions: Machine and human factors

Modern technologies inherently come with unanticipated challenges that can impact treatment durations. Our analysis identified unexpected situations related to the machine or software, ranging from software integration problems to connection hitches. Most issues were resolved quickly with Elekta's support, highlighting the importance of technical assistance, while an error logbook ensured efficient responses to recurring problems.^27^, ^28^, ^29^

Human factors also play a critical role. By refining responsibilities and providing ongoing training for radiation therapists (RTTs), we enhanced task execution and overall workflow efficiency.^30^, ^31^

### 4.3 Safety and special cases

Specific protocols were developed for patients with special needs, such as those with pacemakers, to ensure safety during MR‐guided treatment. Two pacemaker patients were treated despite MRI's relative contraindication due to significant benefits, requiring coordination with cardiology teams and highlighting the importance of rigorous MR safety practices, including staff training for handling potential hazards.^32^

### 4.4 Workflow optimization and efficiency gains

By analyzing the time distribution during MR‐Linac treatment sessions, we gained valuable insights into system efficiency and identified key areas for improvement. In addition to the tips mentioned earlier, we also implemented strategies tailored to specific clinical scenarios to further enhance workflow efficiency and patient care. For instance, we decided to consistently use the ATS workflow for prostate cancer patients, eliminating time lost in the workflow decision process and the co‐registration step. Directly proceeding to the contouring phase, particularly for minor changes, further reduced overall treatment time. Additional steps, such as incorporating functional imaging (e.g., DWI‐MRI), also influenced session duration.

A major milestone improvement was the implementation of a digital briefing sheet, which substantially improved treatment safety and facilitated internal traceability. This tool enabled efficient communication of significant information among team members, ensuring consistency across sessions, especially given the frequent day‐to‐day changes in staff. Continuous evaluation and adaptation of workflows in response to unexpected events remain essential to further optimize treatments and enhance efficiency.

### 4.5 Research opportunities and future directions

The MR‐Linac also serves as a platform for innovative clinical research, exploring motion management, dosimetry variations, and tumor biology thanks to functional MRI, among other possibilities.^12^, ^13^ We focus on developing and using functional MRI (DW and intravoxel incoherent motion [IVIM] MRI) on the MR‐Linac.^33^, ^34^, ^35^ This allows us to capture daily images for precise analysis of the microenvironment and tumor response to radiation, without disrupting the patient's clinical path. Prospective studies initiated by the Institut Jules Bordet, such as STEREOPAC for borderline resectable pancreatic cancer (NCT05083247), and a planned study for rectal cancer, illustrate these opportunities.^36^

## 5 CONCLUSION

Our journey through the first 150 MR‐guided treatments illustrated challenges and unforeseen events encountered with the implementation of this new technology in a radiotherapy department. This represents an opportunity to revisit and optimize the clinical workflow. Carefully analyzing screen recordings was crucial to address and learn from unexpected events. The introduction of mandatory standard operating procedures in the online workflow and the utilization of briefing sheets enhanced our ability to provide high‐quality patient‐centered care. As our understanding of technology continues to evolve, we aim to maintain a dynamic and responsive approach to address new challenges that may arise during MRgRT. Overall, close collaboration and comprehensive training for physicians, physicists, and RTTs are essential.

## AUTHOR CONTRIBUTIONS

**Madeline Michel**: Conceptualization; methodology; data curation; formal analysis; investigation; visualization; writing—original draft; writing—review & editing; funding acquisition; resources. **Zelda Paquier**: Conceptualization; methodology; data curation; formal analysis; investigation; visualization; writing—review & editing. **Christelle Bouchart**: Resources; writing—review & editing. **Akos Gulyban**: Writing—review & editing. **Dirk Van Gestel**: Writing—review & editing. **Nicolas Jullian**: Resources; writing—review & editing. **Sara Poeta**: Writing—review & editing. **Nick Reynaert**: Writing—review & editing. **Philippe Martinive**: Resources; funding acquisition; writing—review & editing; supervision. **Robbe Van den Begin**: Resources; writing—review & editing; supervision. All authors have read and approved the final manuscript and agree to be accountable for all aspects of the work.

## CONFLICT OF INTEREST STATEMENT

The Institut Jules Bordet radiation oncology department is a reference site for Elekta and has an ongoing research agreement with Elekta, which is independent of this work. Otherwise, the authors declare no competing interests.

## Supporting information



Supporting information
