# Supplementary material for: Facilitating 1.5T MR‐Linac adoption: Workflow strategies and practical tips
Source: J Appl Clin Med Phys. 2025 Mar 16;26(5):e70073. doi: 10.1002/acm2.70073 (PMC12059274; doi:10.1002/acm2.70073)

Supplementary material

1. Workflow improvements at each phase and their impact

- **Briefing sheet for each patient**
- **Why it was introduced:** We operate on a rotation system with team members alternating responsibilities throughout the week. Despite holding a weekly meeting to review cases scheduled for the upcoming week, it was challenging to remember all critical details or specific considerations, particularly for those not specializing in certain pathologies. Hesitations or forgetfulness could result in time loss or reduced treatment quality, which is unacceptable. This made the implementation of a concise and effective communication channel, accessible to all team members, an essential necessity.
- **How it works:** It includes imaging protocols, contouring strategies, dose constraints, and daily notes, allowing for verification of patient preparation (e.g., bladder, rectum, stomach) and documentation of deviations.

There are instructions for image acquisition, determined by physicians and executed by RTTs at the MRI console, as well as notes on alternative shorter sequences that may be used if necessary.

The fusion and contouring section provides a comprehensive list of structures requiring adjustment during recontouring, accompanied by specific guidance when applicable. Margin "recipes," as defined by the physician, are also detailed. Margin structures are pre-created offline by the physicist to allow for automatic generation during online contouring. In cases where manual margin adjustments are necessary online, the document provides the original parameters as a reference.

Sections on optimization and desired dose constraints provide further guidance for treatment planning and execution.

A dedicated treatment progress section records key information for each fraction, including the adaptive method used (ATS or ATP). For pelvic cases, bladder filling status (empty, semi-full, full) is documented to aid in selecting a reference fraction if needed. This section also captures any unmet constraints, patient-specific requests, or other critical observations, ensuring continuity and precision throughout the treatment course.

- **Impact:** The briefing sheet established a concise and effective communication channel that became essential for all team members. It was developed to centralize patient-specific information, ensure consistent communication among team members, and standardize MRgRT workflows. It has significantly reduced miscommunication, streamlined daily operations, and saved time across all workflow phases. Additionally, its clear treatment protocols have facilitated the onboarding of new staff and prevented delays when managing patients with multiple OARs requiring attention.
- **Example :** See supplementary material B.
- **“Guide adapt” support structure to re-contour efficiently**
- **Why it was introduced:** After treating several patients, it quickly became evident that recontouring all OARs was excessively time-consuming and not always dosimetrically relevant. Following recommendations from the MR-Linac consortium and observations of treatment plans, the Guide Adapt structure emerged as the optimal solution.
- **How it works:** This involves creating a support structure around the target volume (GTV or CTV or PTV) to streamline re-contouring. It is generated as a margin around the selected volume. A 30mm expansion is typically applied, but this can be adjusted based on specific needs. The structure acts as a boundary to limit adjustments within a defined region, minimizing time spent on re-contouring and focusing on critical areas close to the tumor.
- **Impact:** The Guide Adapt structure was designed to streamline re-contouring by focusing on critical regions near organs at risk (OARs), where precision is most needed, significantly reducing contouring time while ensuring consistent treatment accuracy, particularly in complex cases involving multiple OARs, such as pancreatic cases.
- **Example :** The guide adapt (in red) serves as a boundary beyond which the OARs are either not re-contoured or, if necessary, not contoured precisely (when a volumetric value is required).


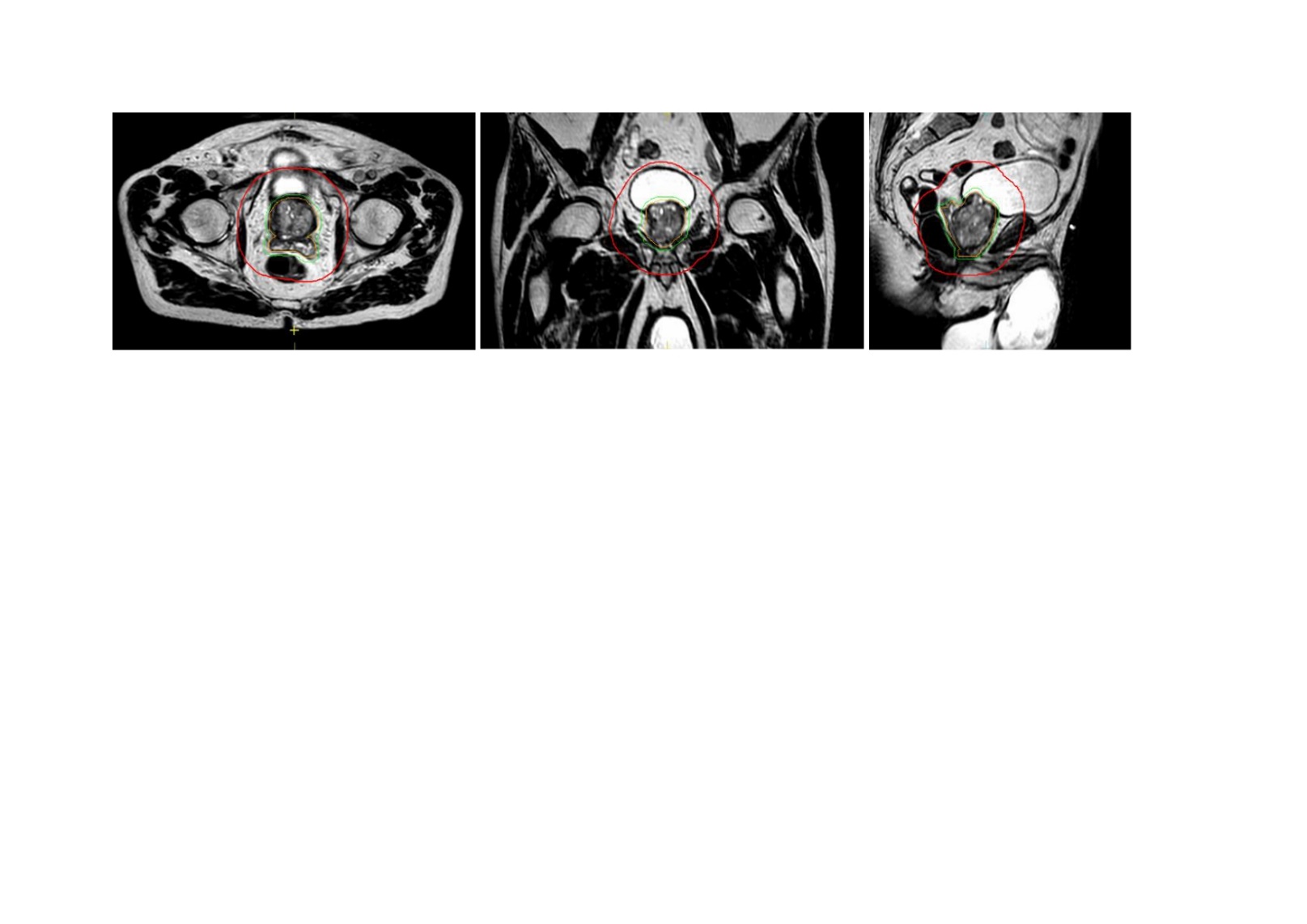


- **Record solutions to previous technical problems (error logbook)**
- **Why it was introduced:** It was introduced shortly after repeated occurrences of problems whose resolution was complex or non-intuitive. Without it, team members encountering an issue often faced two options: spending considerable time figuring out the solution themselves, or contacting other colleagues, hoping they could provide an answer.
- **How it works:** The error logbook includes machine-reported errors, such as those occurring during quality assurance (QA) or treatment sessions. This logbook is available on hospital computers and can be quickly accessed in case of an issue. For first-time incidents, the issue is added to the log, ensuring continuous updates. This is particularly useful when new team members, inexperienced with the Unity system, join the team.
- **Impact:** This facilitates communication about potential issues across the entire team and provides guidance for those encountering an error for the first time or when the resolution is not readily remembered due to its infrequent occurrence. It is also valuable for troubleshooting quality assurance processes and machine setup. While few errors occur during actual treatment, addressing them efficiently minimizes disruptions and prevents unnecessary delays by improving response times.
- **Example :** Extract from the error logbook.

| Error Message | During | Software | Action |
| --- | --- | --- | --- |
| Scan resources not available. A restart is required": It can not start the MRI and demand to restart, but not enough. (The link between software and table is also broken (can not change music level or ventilation from command)) | Start MRI scan (no problem if from SPT) | Philips | Need to lower circuit-breaker/fuse in the cabinet next to Philips cabinet: there are 6-7 fuses to lower in the order of their numbers, then relaunch it in the same order after 10 seconds (see sheet inside the cabinet, but just restart fuses is enough) |
| Issue with the Rx Site in the prescription (missing or incorrect name, discrepancy between Monaco and Mosaiq). | Export from Monaco Online | Mosaiq | Open the patient in Offline Monaco, unapprove the plan, enter the correct name in the Rx Site for the plan of the day, save, and close. In Online Monaco, reopen the patient and approve the plan. |

- **Clean structures before recreating margins**
- **Why it was introduced:** During online contouring, following automatic deformation, some regions may display small stray points or volumes outside their intended boundaries. Removing these can be time-consuming and, more importantly, they might go unnoticed. If the affected region serves as the base for another margin-based volume, it can lead to errors in the newly generated volume, potentially impacting dosimetry.
- **How it works:** Before recreating new margins during the online treatment, ensure all prior structures are cleaned to avoid erroneous data points. This step prevents incorrect dose calculations during re-planning. You simply need to click the button named “EZclean”, and select « clean all structures ».
- **Impact:** Using the "EZclean" function ensures accurate margin generation by removing stray points, preventing errors in derived volumes, and avoiding potential dosimetric issues. This step streamlines contouring and planning by preventing issues that would otherwise require time-consuming corrections, improving overall workflow efficiency.
- **Example :** The "Clean" button is highlighted in red in this image, and the options panel appears in the center of the screen after clicking the button.


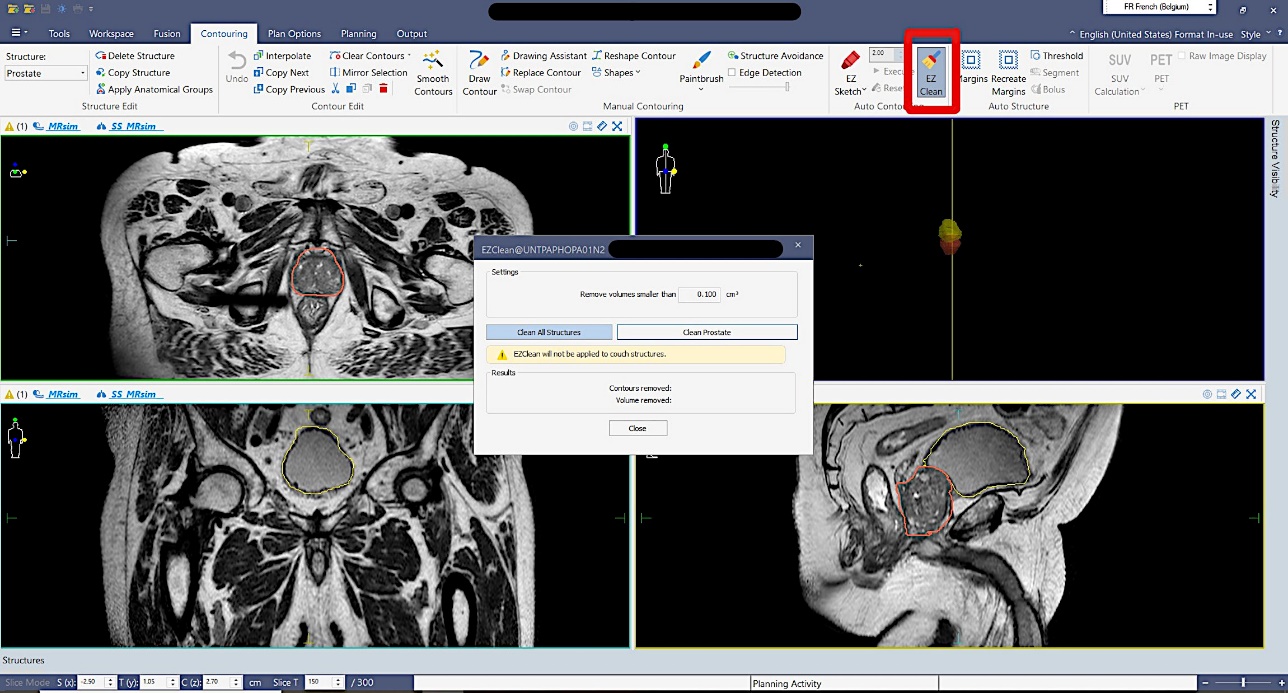


- **Prepare in advance the structures to crop before online planning.**
- **Why it was introduced:** For adjacent structures with a shared interface or border (e.g., prostate and rectum), this approach eliminates the need to contour the same interface twice, once for each structure. Offline, on the simulation images, this is managed using boolean operations. During online contouring, these operations needed to be automated to address the challenge of manually repeating the process each day. This method also applies to margin structures like the PTV.
- **How it works:** Standard margin structures are encoded during reference planning to ensure they are automatically recreated during online contouring. Additionally, structures generated using boolean operations on simulation images are also defined with margins to streamline the process further. To prepare them, we use the briefing sheet, which includes the structures to be cropped and their "recipe" as chosen by the physician. During daily re-contouring, the process is completed by selecting "recreate margin" as the final step.
- **Impact:** Predefined cropping ensures precision and reduces time spent on contouring during treatment, particularly in anatomically complex regions.
- **Example :** The first image illustrates how the physicist prepares the margins offline. The second image shows the button that needs to be pressed online to recreate all the margin structures previously prepared.


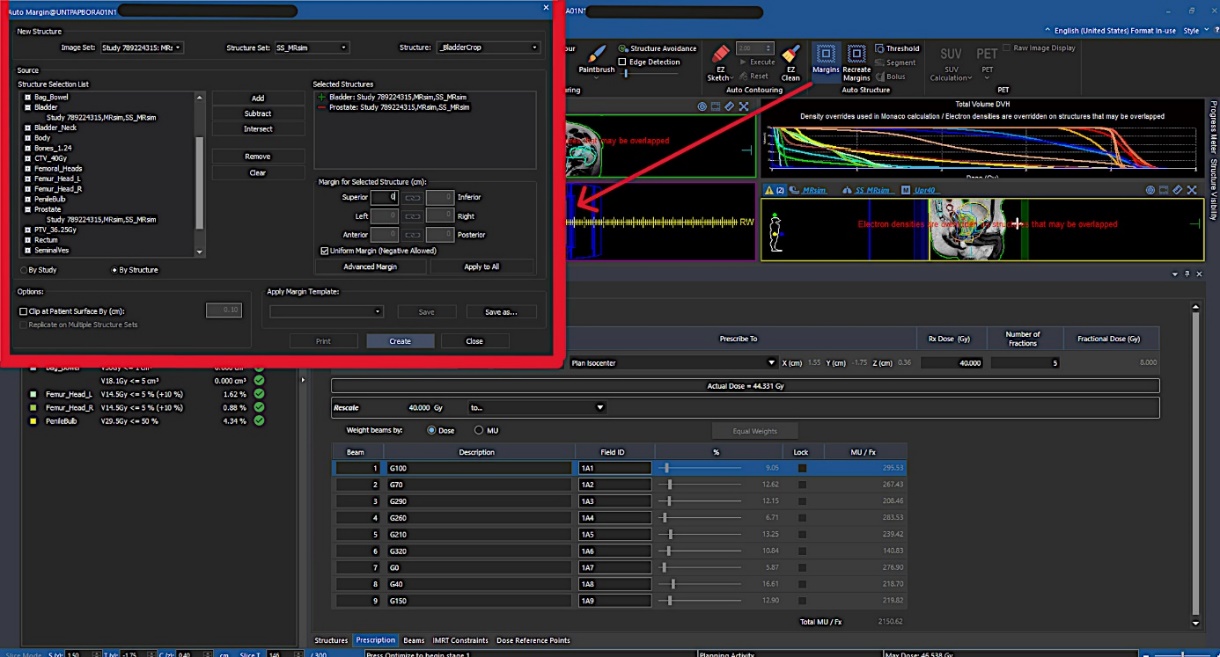


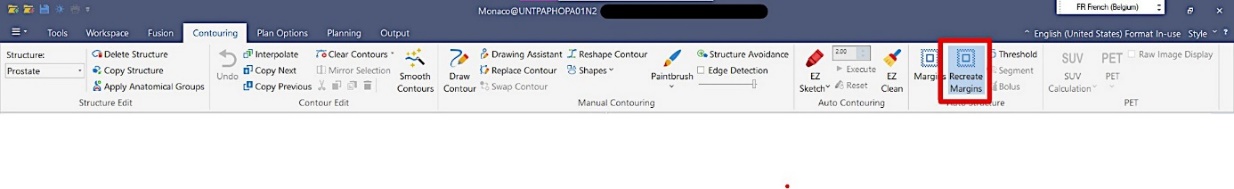


- **Empty the Monaco Online import folder if the last imaging before plan approval was a functional sequence.**
- **Why it was introduced:** There were several instances where we acquired a functional sequence during re-contouring. If the process was quick, we skipped the verification imaging before starting motion monitoring. In this case, when clicking "approve plan" to export to Mosaiq, Monaco would crash, and nothing would be sent. After discussions with Elekta, the source of the error was identified.
- **How it works:** Functional sequences not approved by Elekta (e.g., DWI) should be deleted from the import folder before approving the plan in Online Monaco version 5.51.11 to avoid crashes during the export process. No longer required in version 6.2.2.
- **Impact:** Clearing the import folder prevents workflow interruptions and saves valuable time by avoiding crashes during the plan approval process.
- **Additional screen with baseline contours above the main screen**
- **Why it was introduced:** Initially, the reference contours were displayed on a nearby screen, positioned at a perpendicular desk. While accessible, this setup made it difficult to efficiently cross-check contours with the Monaco interface. By adding a second screen directly above the Monaco workstation, we ensured that both could be viewed side by side, significantly improving workflow and ease of use, especially for less experienced physicians or those unfamiliar with the pathology.
- **How it works:** We added an additional screen directly above the one used for online contouring. This screen displays the reference contours and also provides access to the briefing sheet. This setup allows the physician to quickly verify key details, such as organs to focus on, structures to maintain in rigid mode, or specific case considerations.
- **Impact:** Displaying the patient's baseline contours on a secondary screen provides a quick reference for accurate adjustments during re-contouring. It improves alignment and ensures consistency in contour adaptation. This second screen has reduced contouring time, particularly when the physician on duty was not an expert in the pathology. It also provided reassurance, often eliminating the need to consult the referring physician. This is a non-negligible comfort.
-
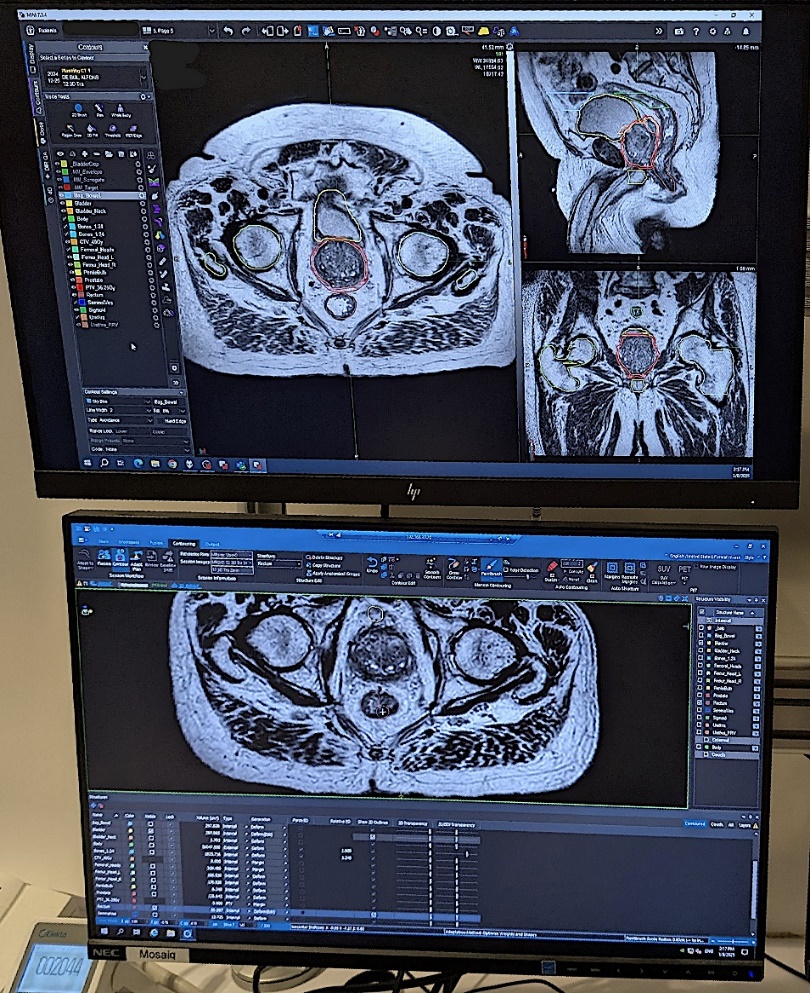
**Example :**

Screen displaying reference contours and the briefing sheet

Main screen

Main screen

- **Consistently use the ATS workflow for prostate cancer patients**
- **Why it was introduced:** Deformable registration generally performs well for prostate cases. By analyzing treatment times during this study, we observed that decision-making between ATS and ATP workflows often introduced unnecessary delays, and in some cases, ATS treatment times were even shorter than ATP. Additionally, in ATP workflows, hotspots exceeding urethral constraints were consistently observed, which could only be mitigated by partially uncovering the PTV/CTV, highlighting a limitation of this approach.
- **How it works:** Based on these findings, we opted to proceed directly with the ATS workflow for prostate cases, which typically require only minimal adaptations. This approach improved both session quality and efficiency.
- **Impact :** Save time during online treatment by eliminating time lost in the workflow decision between ATP and ATS and the co-registration step, while also achieving better respect of constraints.

1. Example of the Briefing sheet for prostate patient.

.

Practically speaking, all elements of this document can be modified if necessary. The sections highlighted in red are the most likely to require adjustments across different pathologies. A color-coding system indicates responsibility for completing each section: blue for physicians, green for physicists, and turquoise for both. Here, the template has been specifically established for prostate cases.

For the “Fusion/contour” part, the order in which the structures should be check are fixed by pathology and was decided after a team concertation. The margins and constraints are also fixed and were determined after a team consensus.

The underlined elements are specific to centers equipped with Comprehensive Motion Management (CMM). For those that do not yet have access, in the "structures stated as margin" table, the first line can be replaced with ".MM" which represents the structure to visually track during beam-on.


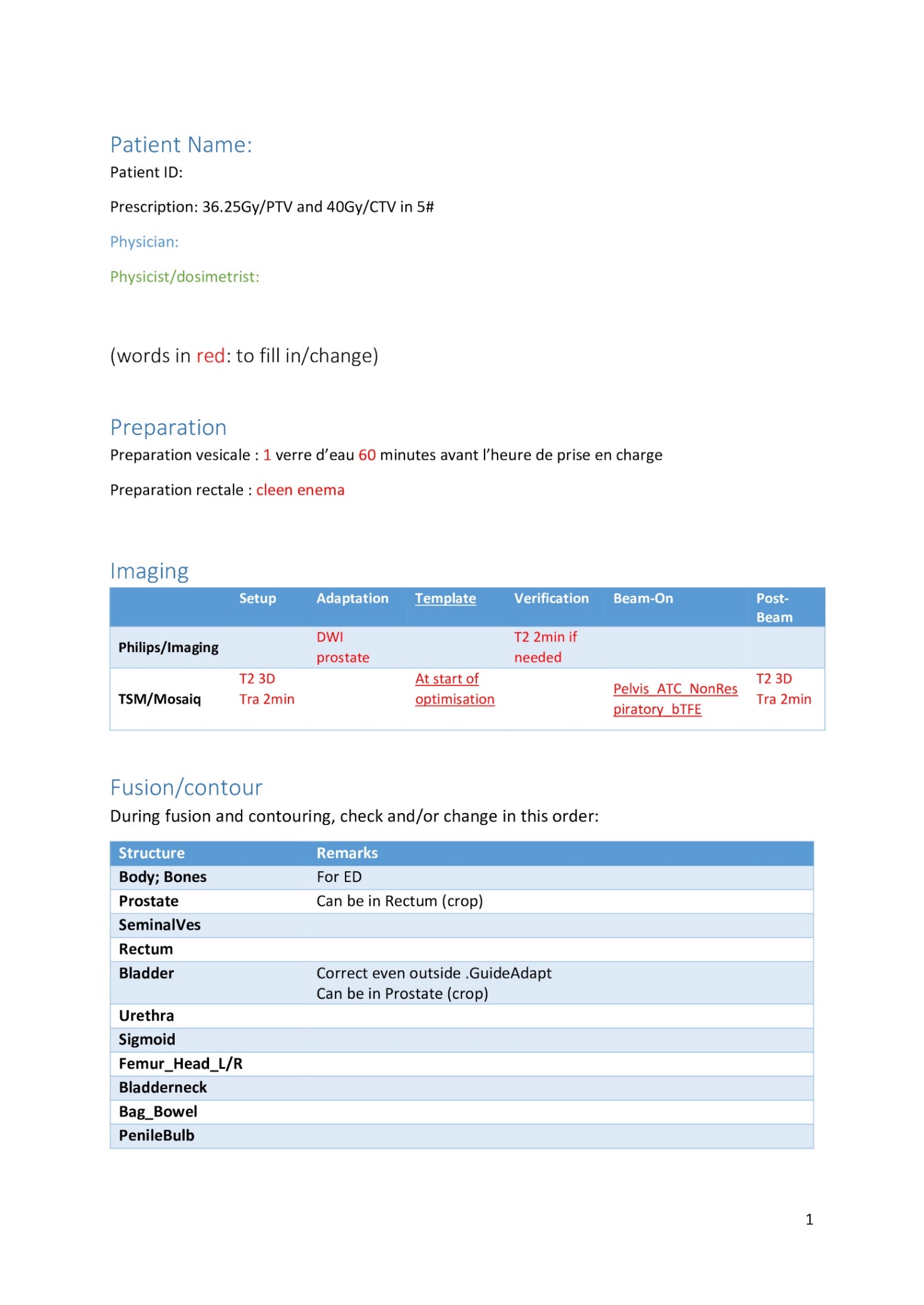


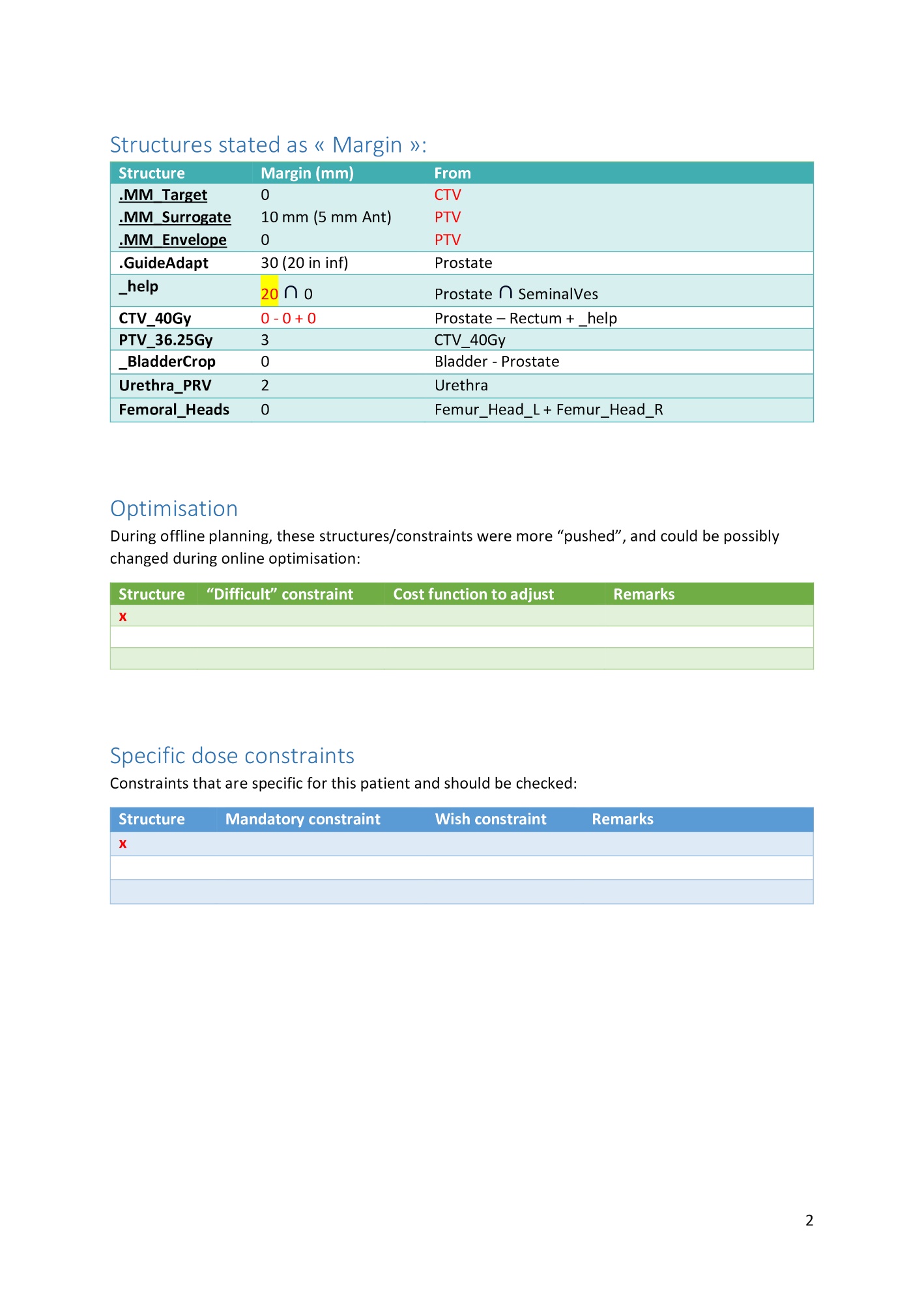


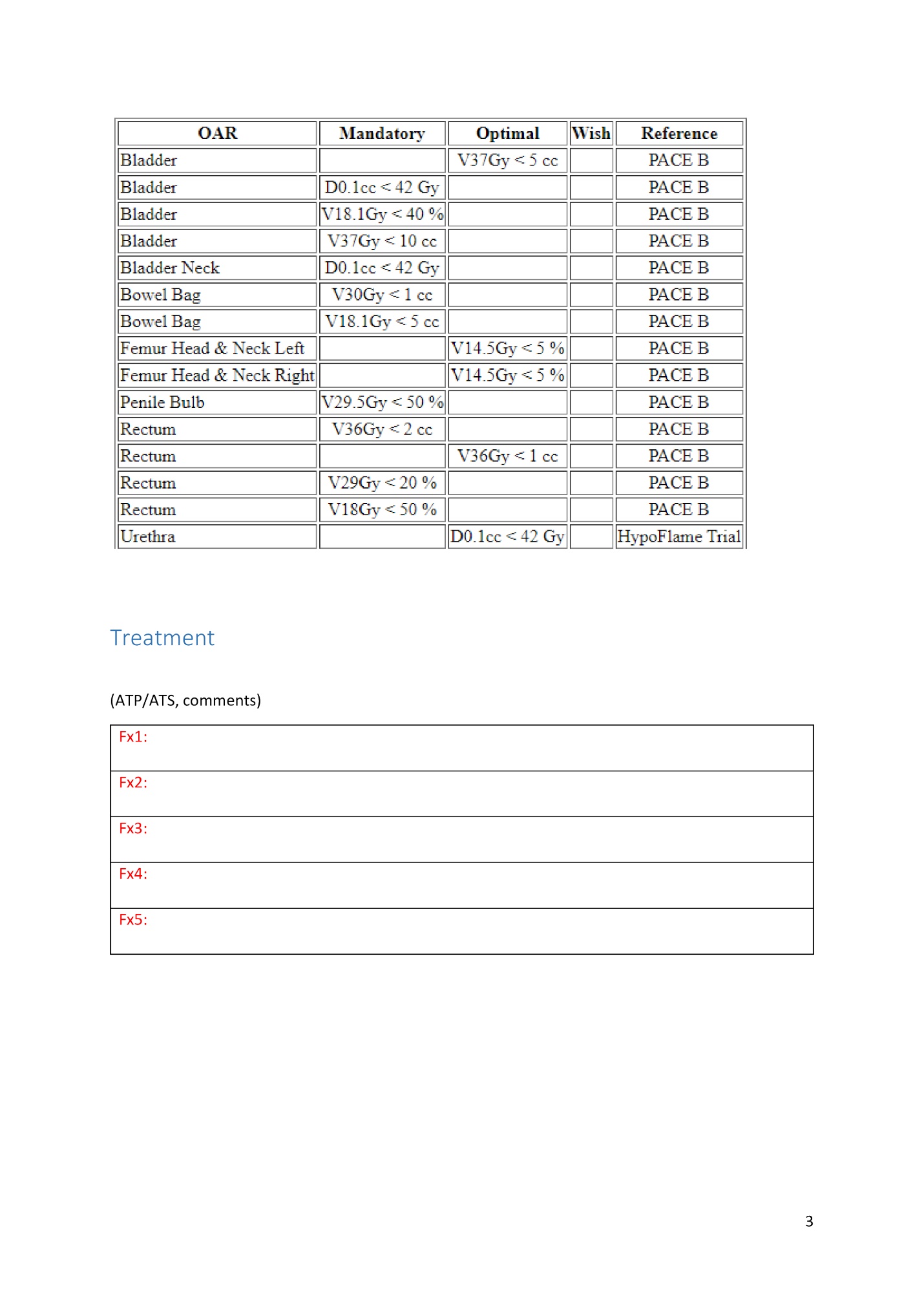

Supplement: Supplementary file 1 — Supporting information [file ACM2-26-e70073-s001.docx]
